# Supplementary material for: Automating multimodal microscopy with NanoJ-Fluidics
Source: Nat Commun. 2019 Mar 15;10:1223. doi: 10.1038/s41467-019-09231-9 (PMC6420627; doi:10.1038/s41467-019-09231-9)
Supplement: Supplementary file 1 — Supplementary Information [file 41467_2019_9231_MOESM1_ESM.pdf]

Automating multimodal microscopy with NanoJ-Fluidics

Almada and Pereira et al.

Supplementary Note 1: Design and assembly
Supplementary Note 2: NanoJ-Fluidics workflows
Supplementary Note 3: Characterisation of the accuracy and precision of NanoJ-Fluidics
Supplementary Note 4: Resolution mapping
Supplementary Note 5: Nanoscale morphological changes between pre- and post-fixation

Supplementary Figure 1. Assembled NanoJ-Fluidics system on a microscope
Supplementary Figure 2. NanoJ-Fluidics pump assembly
Supplementary Figure 3. User Interface
Supplementary Figure 4. Characterization of accuracy and precision of the NanoJ-Fluidics syringe pump array
Supplementary Figure 5. Fourier Ring Correlation (FRC) resolution mapping for Fig. 3 and Supplementary Movie 3 using NanoJ-SQUIRREL
Supplementary Figure 6. Analysis of changes in cell morphology and labelling pre- and post-fixation
Supplementary Figure 7. Fourier Ring Correlation (FRC) resolution mapping for Fig. 4 and Supplementary Movie 4 using NanoJ-SQUIRREL

Supplementary Table 1. Breakdown of the costs of parts for NanoJ-Fluidics arrays
Supplementary Movie 1. Event-driven live-to-fixed imaging with NanoJ-Fluidics
Supplementary Movie 2. Unsupervised live-to-fixed microscopy triggered by mitotic cell rounding
Supplementary Movie 3. Live-to-Fix Super-Resolution Imaging with NanoJ-Fluidics
Supplementary Movie 4. Automated Multiplex Super-Resolution with NanoJ-Fluidics
Supplementary Software 1. NanoJ-Fluidics installation file

Supplementary Note 1: Design and assembly

Design and capabilities. The NanoJ-Fluidics system is designed primarily for the sequential exchange of liquids in a glass-bottom cell culture dish (or equivalent supports, such as Attofluor cover slip holders for example). It consists of several parts: Lego based syringe pumps responsible for delivering reagents to the sample (Fig. 1a and Supplementary Fig. 2a); electronics responsible for controlling the pumps (Supplementary Fig. 2b-d); a peristaltic pump responsible for removing reagents from the sample (Fig. 1b and Supplementary Fig. 2d,g); fluid handling disposable components (Fig. 1b and Supplementary Fig. 2e-f). A complete break-down of the components and indicative pricing are shown in Table 1. The syringe pumps are designed to accommodate syringes of any size, to be cost-effective and simple to assemble. A syringe pump unit uses a simple gear and actuator system to convert the fast motor motion into a smooth and slower linear motion, to enable consistent fluid flow. The result is that the flow-rate will depend on the gears, motor speed and the syringe's internal diameter. A detailed description of the NanoJ-Fluidics workflows and characterisations can be found in Supplementary Note 2 and 3 respectively.

Both the syringe pump array (for media injection) and peristaltic pump (for media removal) are digitally run using an Arduino UNO micro-controller and Adafruit Motorshield digital-to-analogue motor control. The Motorshield is an additional electronic board that connects to the Arduino controller, enabling it to run up to 4 Lego motors. An Arduino can be stacked with up to 32 Motorshields, enabling a single board to control up to 128 pumps, which should be sufficient for most NanoJ-Fluidics applications. We provide custom open-source firmware that enables the Arduino-based electronics to be programmatically controlled by a connected computer. We also provide a Java-based graphical user interface (GUI) for simple control of the fluidics sequences (Supplementary Fig. 2).

The software interface can be set to automatically perform a sequence of liquid injection and liquid removal steps (Supplementary Fig. 3) allowing an entire sample fixation or labelling protocol to be carried as shown in Fig. 2-4.

Assembly. The steps to prepare a complete functioning NanoJ-Fluidics system (Supplementary Fig. 1 and 2) consist of assembling the pump array, controller, and wiring them together (Supplementary Fig. 2a-d). Once the array and controller have been assembled, each experiment only requires the lab-ware component to be prepared anew (Supplementary Fig. 2e-g). A full step-by-step guide for assembly and preparation can be found in https://github.com/HenriquesLab/NanoJ-Fluidics/wiki.

NanoJ-Fluidics can also be used with more traditional microfluidics devices, such as polydimethylsiloxane (PDMS) chips, however we have focused on providing a series of protocols using off-the-shelf labware (e.g. glass-bottom cell culture dishes). This approach makes the framework more accessible to laboratories that do not otherwise have the facilities to produce PDMS devices. When using culture dishes the primary use of the system is to reproduce many laboratory protocols including cell fixation, immunolabeling, drug treatment, among others.

Software interface and experiment automation. Control of the NanoJ-Fluidics pump array can be achieved in any of three ways: by using the provided Java-based GUI (Supplementary Fig. 3); RS232 commands to the Arduino board; or by using the Application Programming Interface (API). A description of these is available at https://github.com/HenriquesLab/NanoJ-Fluidics/wiki.

The RS232 command scheme enables researchers with programming experience to run the controller directly in their own software packages. However, the GUI was designed to enable any user to directly run any number of Arduino controllers and pumps, as well as design a sequence of steps associated to an experimental protocol (Supplementary Fig. 3).

NanoJ-Fluidics is controlled by a set of Java classes described in the API. For example, these can be used to enable a protocol generated in the GUI to be programmatically started and manipulated by outside applications, such as a microscope control software like μManager [1].

Supplementary Note 2: NanoJ-Fluidics workflows

The NanoJ-Fluidics Lego syringe pumps are accurate and precise for a large number of applications. It was however specifically designed to be optimal in protocols involving multiple full replacements of the sample medium. This is achieved by aspirating the sample medium using a single peristaltic pump before loading a specific medium using one of the Lego syringe pumps from the array. As a consequence of this experimental design, the workflow has a high tolerance to errors in accuracy and precision since the concentrations applied to the sample are determined at the set-up stage. Also, with this set-up, it is easy to ensure that the sample will not be left to dry or overflown with liquid.

As with any other syringe pumps, the first step is to load the liquid to be dispensed. This can be done in 3 different ways as shown in Supplementary Fig. 4a:

- "Overflow Loading mode" – which is the best option to deliver the full liquid content loaded into a pump module, this entails the introduction of an additional volume of air when loading the syringe and subsequently setting the pump to inject the equivalent to the liquid plus air volume. This will lead to liquid content be flushed out in its entirety and dispensed onto the sample. This mode may be used when volumes of reagents accessible to the researchers are limited or if these volumes need to be added with high precision (see Supplementary Note 3 and Supplementary Note 3)
- "Tube Loading mode" – the most common option. This entails loading the syringe through the tubing ensuring that both the tubing and the syringe are fully filled with the liquid of interest. This load allows for accurate multiple consecutive uses of the pump for sequential protocol (such as most protocols described in this paper);
- "Syringe Loading mode" - entails loading the syringe with the liquid of interest and then connecting it to the tubing, this option is less accurate as the first injection will be influenced by the dead volume corresponding to the tubing. Nonetheless, if this is accounted for, the following injections will be accurate, as the tubing will be filled. This mode is slightly easier to set up and can be efficient for large volume change such as washes, but we do not recommend using it when volumes need to be accurately injected.

For highest accuracy and precision, we strongly recommend for reagents to be prepared and mixed at the correct concentrations before being loaded into individual pumps. In particular, for single injections, the "Overflow Loading mode" should be used. This permits the injection of valuable reagents with virtually no waste (Supplementary Note 3). Additionally, the modular nature and design pliability of the Lego syringe pumps allow to place a syringe pump on the microscope stage and easily reduce the tubing length and the corresponding dead volumes to as low as 15 µL (estimated from a 0.2 mm inner diameter tubing and a 10 cm long tubing).

Supplementary Note 3: Characterisation of the accuracy and precision of NanoJ-Fluidics

In-depth characterization of NanoJ-Fluidics was performed in order to determine the variability across Lego syringe pumps, syringes sizes and injected volumes (Supplementary Fig. 4). To characterise the Lego syringe pump array we dispensed specific volumes of MilliQ water into a container sitting inside an analytical scale (Sartorius Cubis®, 0.1 mg ± 0.0003 mg) and determined the weight, which correlates directly with the dispensed volume. To compare the dispensed volume and the determined weight ordinary one-way ANOVA was used (using a confidence interval of 99%). The characterisation was done in both “Tube Loading mode” and “Overflow Loading mode” (see Supplementary Fig. 4a).

We first determined if there was variation across different syringe pumps in the NanoJ-Fluidics array. For this purpose, we characterised three randomly-chosen syringe pumps, “Wasp”, “Bee” and “Hornet” loaded with a fully filled 2 mL syringe (MilliQ water). We dispensed the full 2 mL of liquid in 200 µL steps (10 steps), doing this full process five times (Reload 1 to 5) and determined the accuracy and precision of the individual syringe pumps. Although each pump has an individual bias compared to the nominal injection value (as observed by the difference between the mean of the injected volumes and the nominal volume), for the test performed this offset did not vary more than 2.5% between the tested pumps (“Wasp” 201.6 ± 3.7 µL , “Bee” 195.8 ± 4.6 µL and “Hornet” 199.4 ± 3.2 µL, where error is calculated as the standard deviation across N=50 repeats, Supplementary Fig. 4b). This highlighted that different pumps are performing nearly identically. The offset to the nominal volume can be robustly minimised by calibrating each pump individually (Supplementary Fig. 4d). We also present a method for such calibration in the Wiki (https://github.com/HenriquesLab/NanoJ-Fluidics/wiki/Calibrating-pumps).

We then inquired if the accuracy and precision of volume dispensing into the sample would be dependent on the load of the syringe. For this analysis we plotted the aforementioned experiment in function of the total volume injected (Supplementary Fig. 4c). We determined that there is no significant accuracy or precision differences between the different syringe loads. The high accuracy and precision are independent of syringe volume (Supplementary Fig. 4e), although for very large syringe volumes (> 20 mL), the calibration may need to be redone. However, such large volumes are only required for media change or washes where an accurate volume is not essential.

Then, we determined the minimum volume that could be accurately injected into the sample using NanoJ-Fluidics. For this purpose, we used a calibrated pump and the syringes that hold smaller volumes (1 and 2 mL) and determined how the injected volume correlated with the nominal volumes across a range of injected volumes with volumes as small as 3 µL (Supplementary Fig. 4f). We determined that NanoJ-Fluidics is accurate and precise to < 5% of the nominal volume for volumes > 20 µL (1 mL syringe: 20.96 ± 0.17 µL; 2 mL syringe: 19.56 ± 1.49 µL, where error is calculated as the standard deviation across N=5 repeats). For volumes above 20 µL, the NanoJ-Fluidics syringe pumps show good accuracy and precision, as it is evident from Supplementary Fig. 4f.

Finally, to test the reliability of the overflow method, we first collected 100 µL with a Gilson P100 pipette and weighed it to accurately define the picked volume and take into account the imprecision due to the pipetting itself. We then injected that volume into the tubing, followed by a small volume of air (< 100 µL). The reagent was then pushed out of the tubing and onto the weighing scale using the syringe pumps, by infusing the equivalent volume to a 500 µL injection, using a 2mL syringe. This paired weight measurement (pipetted weight vs. infused weight) was performed 10 times. Comparing the overflow method (98.96 ± 1.3 µL, where error is calculated as the standard deviation across N=10 repeats) and the use of research-grade pipettes (99.73 ± 0.9 µL, where error is calculated as the standard deviation across N=10 repeats) resulted in a mean of difference between sample means (μ_{M1-M2}) of 0.77 µL with a standard error (SE) of 0.48 µL, demonstrating the accuracy equivalence of the methodologies.

Supplementary Note 4: Resolution mapping

To estimate the local resolution achieved in the super-resolution renderings associated with this work, we carried out analysis using the Fourier Ring Correlation method [2], recently modified in NanoJ-SQUIRREL [3] to generate a resolution map. We ensured that the estimations of resolution are not affected by the FRC measurements obtained outside of the cells (background) by defining the in-cell resolution, estimated by masking the regions outside the cell in the FRC map. Supplementary Fig. 5 shows that the best resolution achieved for live-cell imaging with SRRF is 172 nm, and 43 nm for fixed-cell imaging with STORM. Supplementary Fig. 7 shows that the best resolution achieved in the DNA-PAINT channels is [illegible]67 nm, whereas for the actin STORM channel an estimated resolution of 97 nm was obtained.

Supplementary Note 5: Nanoscale morphological changes between pre- and post-fixation

To verify that there were no major alterations in cell morphology during fixation, we compared images of the same cell immediately before and after fixation. Supplementary Fig. 6a shows pre- and post-fixation images for HILO imaging of the cell shown in Fig. 3, and Supplementary Fig. 6b shows these changes for SRRF reconstructions of the same region. Overlaying both the SRRF and HILO images pre- and post-fixation shows that the morphology of the cell remains largely constant during fixation. There is movement of the bright filaments within the cell body and filopodia at the cell periphery on a sub-micron scale (highlighted by arrows on Supplementary Fig. 6b - Merge). This degree of movement is comparable to or smaller than the frame-to-frame movement shown in Supplementary Movie 3. There is also a loss of fluorescence intensity during fixation in the top left portion of the region shown on the left-hand side of the difference map (Supplementary Fig. 6a), as highlighted by the large bright patch (yellow, corresponding to live-cell information).

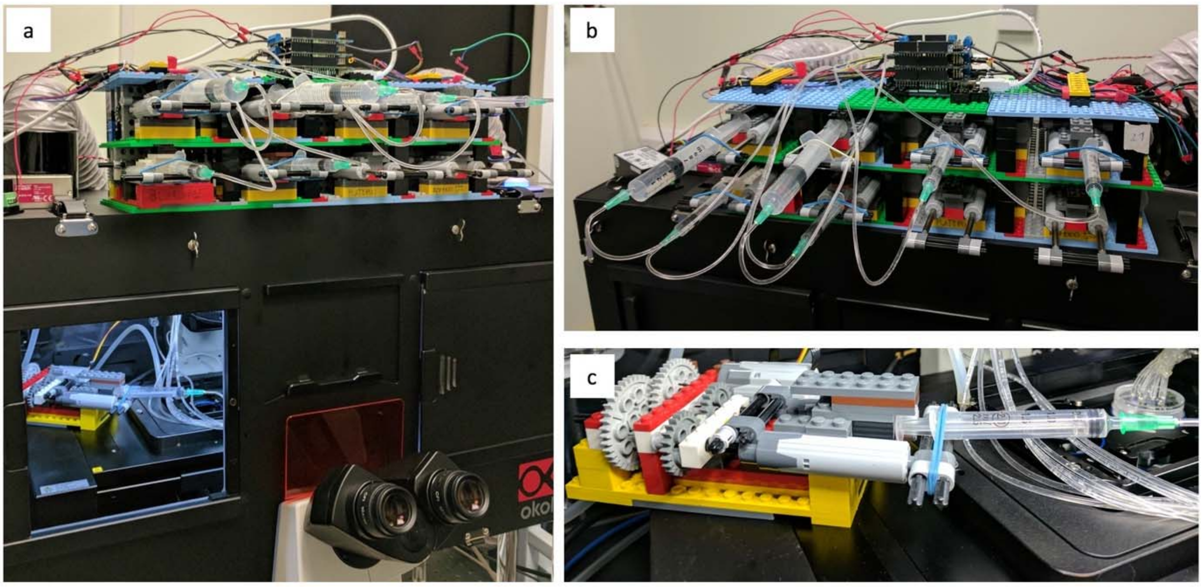

Supplementary Figure 1. Assembled NanoJ-Fluidics system on a microscope. a) View of assembled pump array with syringes loaded on top of a Nikon N-STORM microscope, an individual syringe pump unit sits inside the incubator and is kept at 37°C in the microscope incubator, allowing the use of reagents equilibrated at the same temperature as the sample, such as fixatives. b) Top view of the syringe pump array. c) Zoom into the individual syringe pump unit inside incubator.

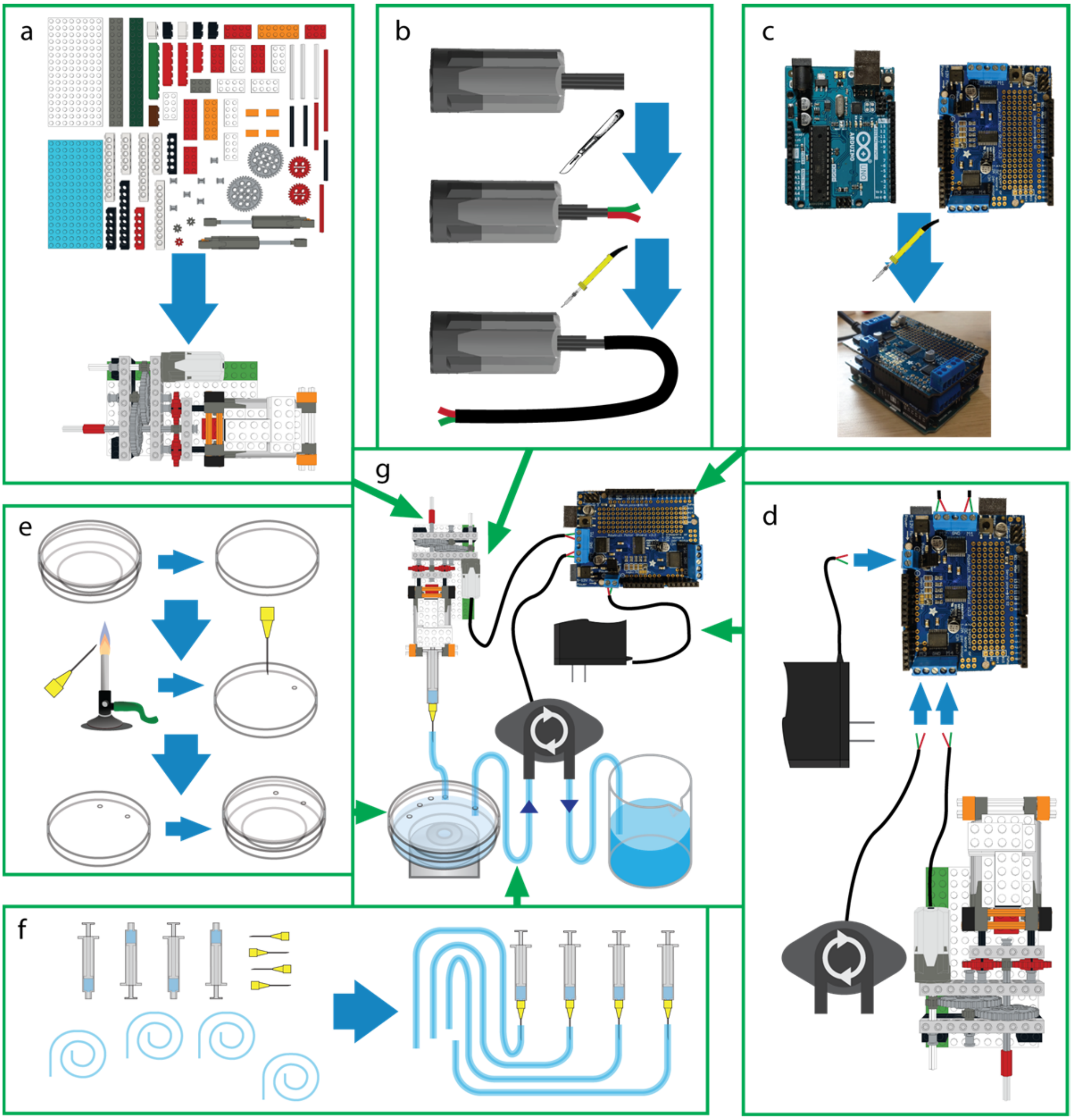

Supplementary Figure 2. NanoJ-Fluidics pump assembly. There are 4 steps to assemble a NanoJ-Fluidics system (a-d) and 2 steps to prepare an experiment (e,f). a) Build the syringe pumps from bricks. b) Assemble the electronic controller. c) Prepare the motor cables for wiring. d) Connect the pumps and power supply to the controller. e) Prepare the syringes. f) Prepare lid of the cell culture dish. g) Thread syringes on the dish lid and mount syringes on pumps.

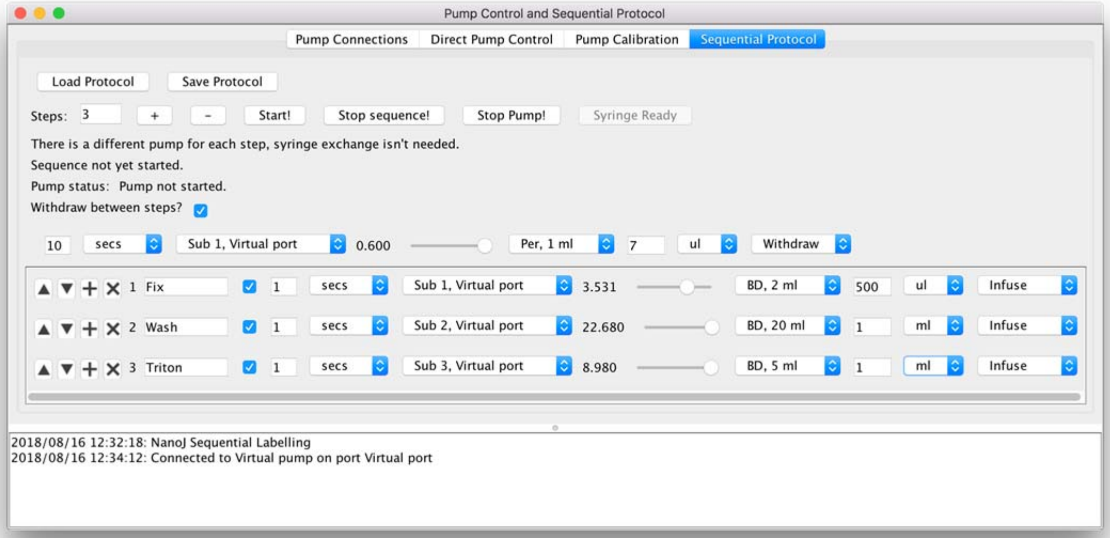

Supplementary Figure 3. User Interface. Screenshot of the NanoJ-Fluidics sequential control user interface.

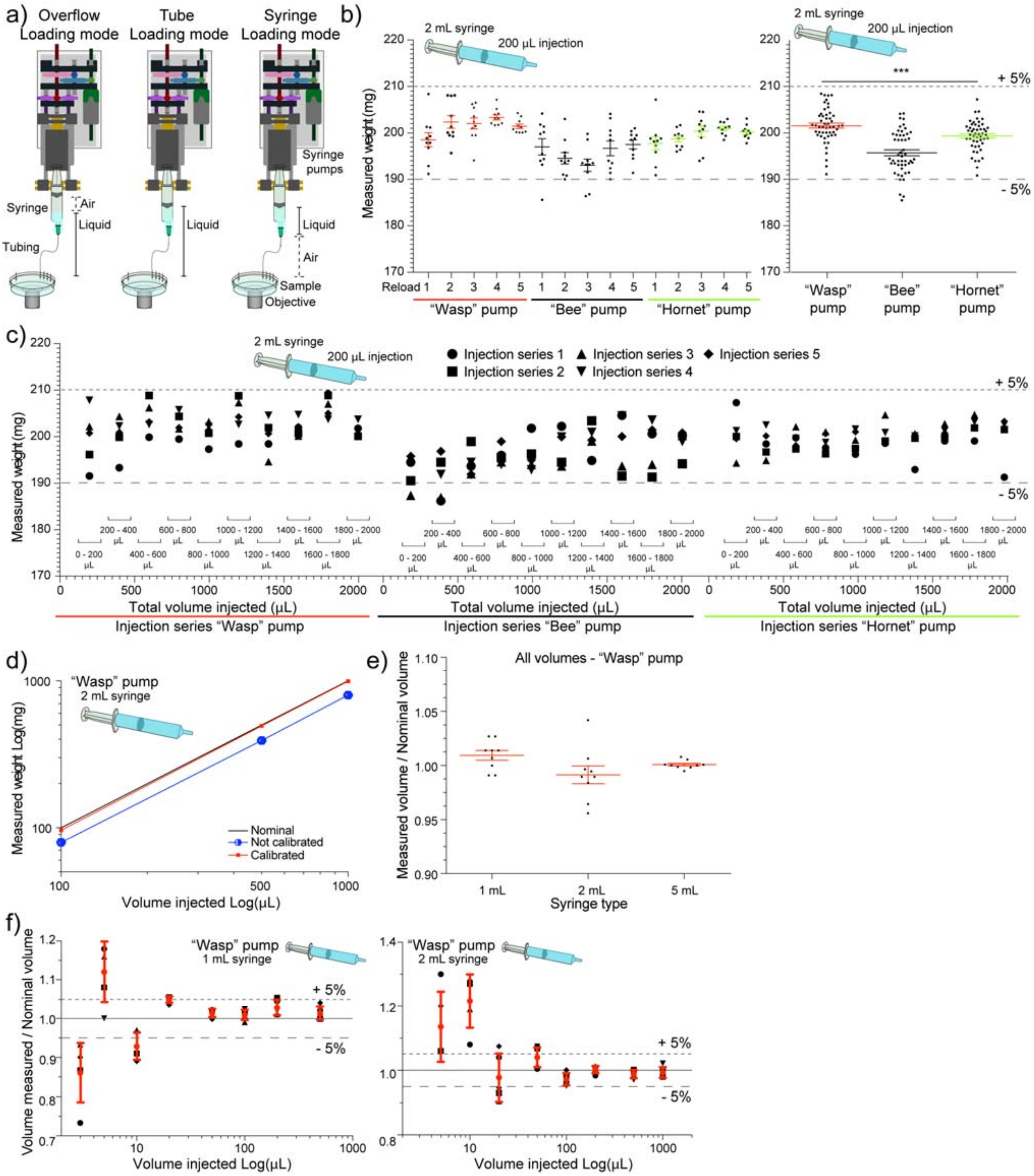

Supplementary Figure 4. Characterization of accuracy and precision of the NanoJ-Fluidics syringe pump array. a) Different modes of liquid loading into the sample using the syringe pump: Overflow loading mode, Tube loading mode and Syringe loading mode. b) Accuracy and precision measurement across (non-calibrated) pumps. The left panel shows all data from 5 reloads on 3 independent pumps (N=10 repeats per re-load). The right panel shows a collated data from all reloads (N=50 repeats per pump, p<0.001 with 99% confidence interval). c) Accuracy and precision measurements depending on the syringe load for 3 independent (non-calibrated) pumps. N=5 repeats of complete infusion, for 3 independent pumps. d) Comparison of a calibrated and a non-calibrate syringe pump across different volumes. e) Ratio of measured volume to nominal volume for the "Wasp" pump after calibration of the pump (N=9 repeats for each volume). f)

Accuracy and precision measurements across different volumes on a calibrated pump to determine the minimum volume that can be accurately injected (N=5 repeats per volume injected). All the graphs display as lines the mean plus or minus the standard error of the mean (SEM) except in f) where the lines correspond to the mean plus or minus the standard deviation (SD).

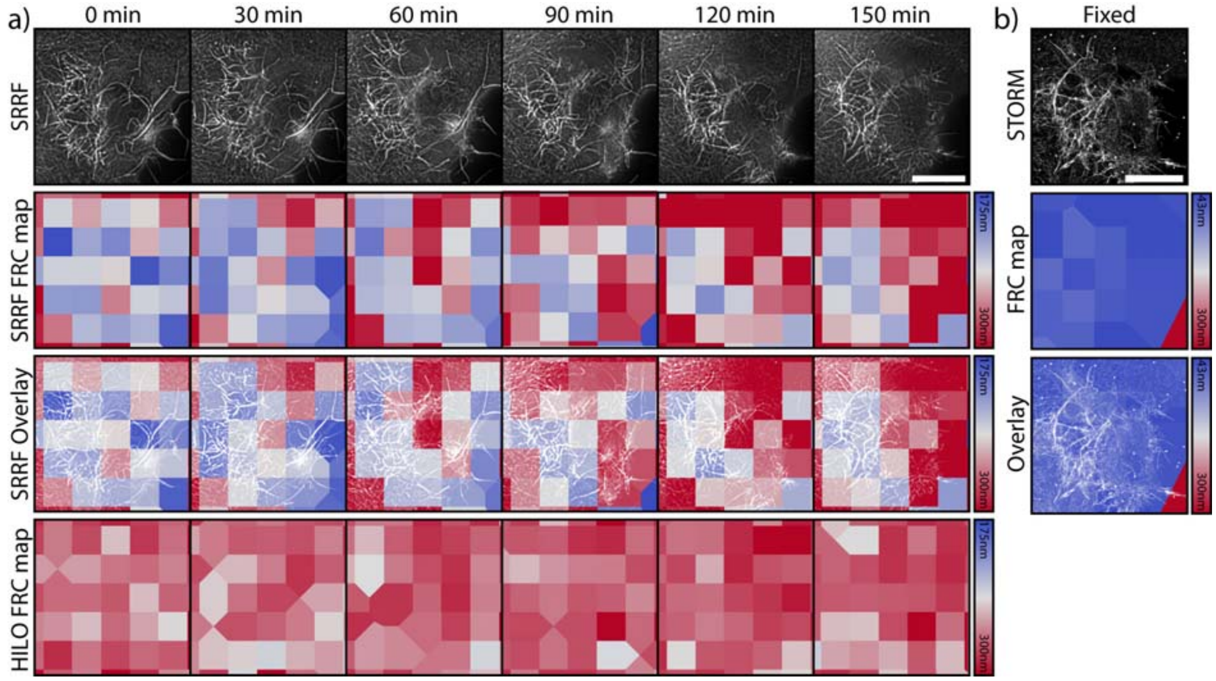

Supplementary Figure 5. Fourier Ring Correlation (FRC) resolution mapping for Fig. 4 and Supplementary Movie 3 using NanoJ-SQUIRREL. a) Individual live-cell SRRF frames at different time-points pre-fixation (SRRF), equivalent FRC map (SRRF FRC map), overlay between SRRF frames and the corresponding FRC map (SRRF overlay) and FRC map for the corresponding diffraction-limited HILO image (HILO FRC map); b) Individual STORM rendering acquired post-fixation (top); equivalent FRC map (middle); overlay between STORM frame and FRC map. Resolution maps calculated through NanoJ-SQUIRREL [3]. All scale bars are 10 μm.

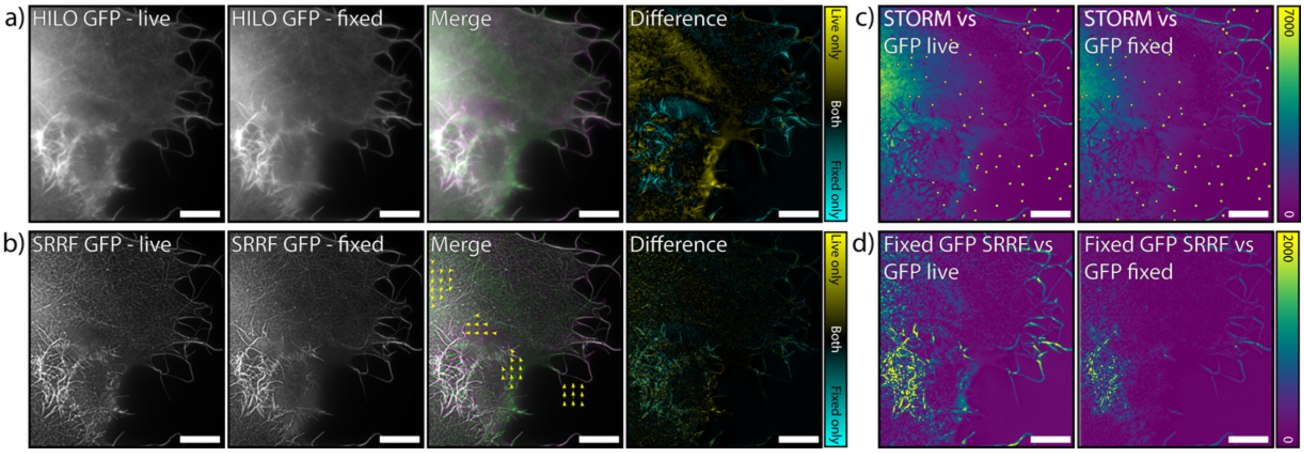

Supplementary Figure 6. Analysis of changes in cell morphology and labelling pre- and post-fixation. a) Comparison of the distribution of GFP-labelled Utrophin in the cell shown in Fig. 4 and Supplementary Movie 3, imaged in HILO. The last timepoint pre-fixation ('HILO GFP - live') and an image post-fixation of the same region ('HILO GFP - fixed') are shown. 'Merge' shows an overlay of the live (green) and fixed (magenta) images. 'Difference' shows the result of subtracting the fixed image from the live image. b) As in (a), except using the SRRF reconstructions of the data. The yellow arrows in 'Merge' show parts of the cell which moved during fixation as measured using the elastic channel registration tool in NanoJ-SQUIRREL. c) Error maps generated between the (fixed) STORM reconstruction of actin in this cell when using the live-cell GFP HILO image (left) or the fixed-cell GFP HILO image (right) as a reference (0 to 7000). d) Error maps generated between the SRRF reconstruction of GFP in the fixed cell when using the live-cell GFP HILO image (left) or the fixed-cell GFP HILO image (right) as the reference (0 to 2000). Scale bars = 10 µm.

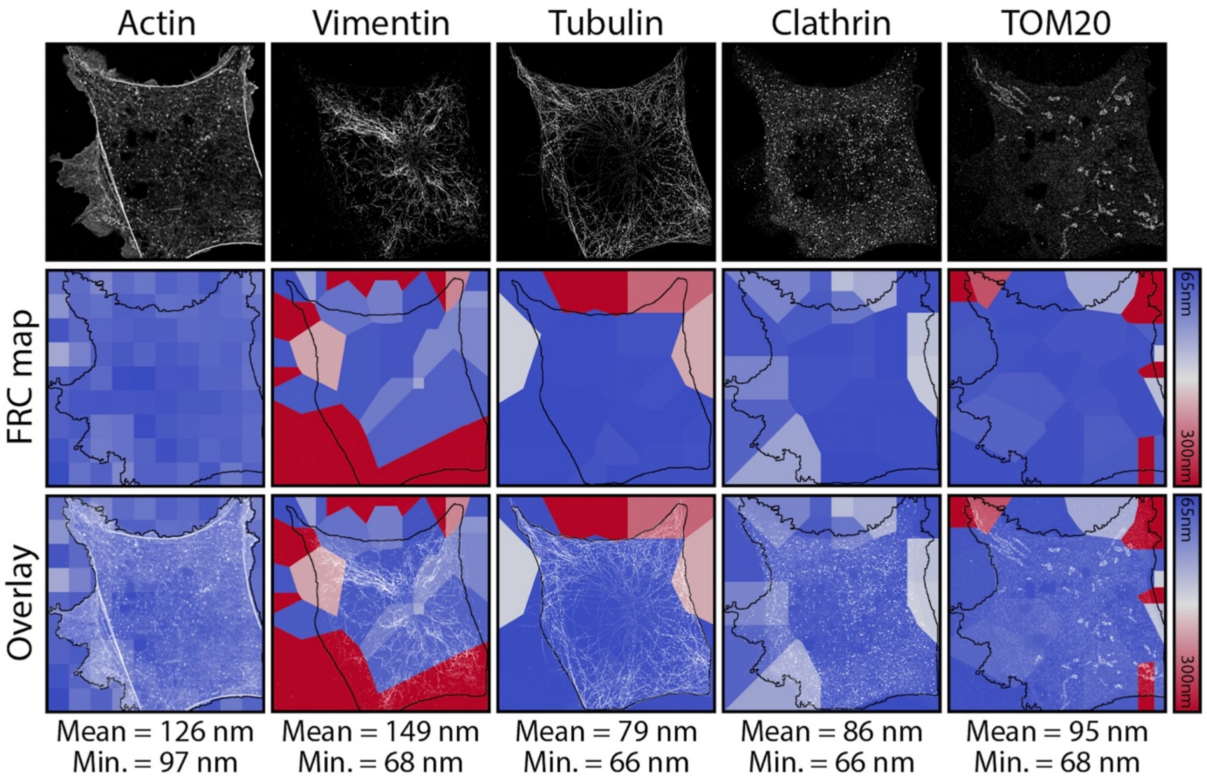

Supplementary Figure 7. Fourier Ring Correlation (FRC) resolution mapping for Fig. 5 and Supplementary Movie 4 using NanoJ-SQUIRREL. Individual DNA-PAINT super-resolution renderings (top-row), equivalent FRC map (middle-row) and overlay between DNA-PAINT images and the corresponding FRC map (Overlay, lower-row). Resolution maps calculated through NanoJ-SQUIRREL. Black outlines in middle and bottom rows indicate the cell shape mask used for calculation of mean and minimum resolutions across each channel.

Sub-system	Parts	Supplier	Supplier code	1 pump	4 pumps	8 pumps
Electronics	Motor shield	Digi-Key	1528-1187-ND	£ 15.50	£ 15.50	£ 31.00
	Arduino	CPC Farnell	SC14867	£ 9.95	£ 9.95	£ 9.95
	USB Cable	CPC Farnell	CS30619	£ 1.16	£ 1.16	£ 1.16
	9V power supply	CPC Farnell	PW04134	£ 8.45	£ 8.45	£ 8.45
	Jumper Wires	CPC Farnell	SC13053	£ 3.99	£ 3.99	£ 3.99
	Stacking headers	CPC Farnell	SC12964	£ -	£ -	£ 2.24
Peristaltic pump	-	CPC Farnell	MC02068	£ 26.83	£ 26.83	£ 26.83
Rubber bands	-	Amazon	B000NMBPQS	£ 2.19	£ 2.19	£ 2.19
Lego pump	Lego store and Brick Owl Lego marketplace			£ 32.00	£ 128.00	£ 256.00
Lego multiplex supports	Lego store and Brick Owl Lego marketplace			£ -	£ 2.00	£ 4.00
Lego vertical array supports	Lego store and Brick Owl Lego marketplace			£ -	£ -	£ 3.20
			**SYSTEM TOTAL**	**£ 84.57**	**£ 182.57**	**£ 318.01**
Labware	Syringe	VWR	613-3911	£ 0.18	£ 0.70	£ 1.40
	Needles	VWR	613-2011	£ 0.06	£ 0.25	£ 0.50
	Tubing (metre)	Cole Parmer	WZ-06460-10	£ 2.62	£ 10.49	£ 20.97
			**USE COST TOTAL**	**£ 2.86**	**£ 11.43**	**£ 22.87**

Supplementary Table 1. Breakdown of the costs of parts for NanoJ-Fluidics arrays. Prices are estimated at the time of writing of this article.

Supplementary References

1. Edelstein, A. D. *et al.* Advanced methods of microscope control using uManager software. *J. Biol. Methods* **1,** 1–10 (2014).
2. Nieuwenhuizen, R. P. J. *et al.* Measuring image resolution in optical nanoscopy. *Nat. Methods* **10,** 557–62 (2013).
3. Culley, S. *et al.* Quantitative mapping and minimization of super-resolution optical imaging artifacts. *Nat. Methods* **15,** 263–266 (2018).
