# Supplementary material for: Automating multimodal microscopy with NanoJ-Fluidics
Source: Nat Commun. 2019 Mar 15;10:1223. doi: 10.1038/s41467-019-09231-9 (PMC6420627; doi:10.1038/s41467-019-09231-9)
Supplement: Supplementary file 3 — Description of Additional Supplementary Files [file 41467_2019_9231_MOESM3_ESM.docx]

**Description of Additional Supplementary Files**

File Name: Supplementary Movie 1

Description: **Event-driven live-to-fixed imaging with NanoJ-Fluidics.** Movie corresponding to Fig. 2. Left: colour coded time projection of a stitched mosaic (17x17 individual regions), following RPE1 cells stably expressing zyxin-GFP undergoing mitotic rounding. Upon enough cells rounded (event detection) cells were fixed and immunolabelled for active β1-integrin and stained for actin and DNA. On the top right corner, overlay of zyxin-GFP in live cells with active β1-integrin after fixation. First and last regions correspond to cells not undergoing mitotic rounding, whereas all remaining regions display RPE1 cells rounding and showcase the colocalization of active β1-integrin (post-fixation staining) with focal adhesions (zyxin-GFP). Bottom right corner, overlay of active β1-integrin, phalloidin and DAPI staining after fixation. Scale bar corresponds to 0.5 mm.

File Name: Supplementary Movie 2

Description: **Unsupervised live-to-fixed microscopy triggered by mitotic cell rounding.** Movie corresponding to Fig. 3. First, time-course imaging of 6 different HeLa HRTG showing the mEGFP-α-Tubulin channel with 5 min time interval. The corresponding masks and circularity (C) values obtained from the automated image analysis are shown. A delay between cell rounding and pump activation is always present due to the time necessary for a sufficient number of fields-of-view to trigger and the 15 min delay introduced from event triggering. See Supplementary Note 5 for detailed explanation of how triggering was performed. Then, their corresponding 3D stacks are shown sequentially for different z-position with 0.5 μm interval. Scale bars correspond to 10 μm.

File Name: Supplementary Movie 3

Description: **Live-to-Fix Super-Resolution Imaging with NanoJ-Fluidics.** Movie corresponding to Fig. 4. HILO and HILO-SRRF live imaging of transiently transfected COS7 cells expressing UtrCH-GFP. After live-imaging the same cells were fixed and stained with phalloidin-AF647 for STORM imaging. All steps were performed using the NanoJ-Fluidics syringe pump array without taking the sample from the microscope. Scale bars correspond to 10 μm.

File Name: Supplementary Movie 4

Description: **Automated Multiplex Super-Resolution with NanoJ-Fluidics**. Movie corresponding to Fig. 5. STORM imaging of COS7 cells stained with phalloidin-Atto488, and DNA-PAINT imaging of vimentin, TOM20, tubulin and clathrin the same cell. Imaging buffer exchange steps were performed using the NanoJ-Fluidics syringe pump array directly on the microscope stage. Scale bars corresponds to 10 μm.

File Name: Supplementary Software 1

Description: **NanoJ-Fluidics installation file.** Zip file containing the NanoJ-Fluidics stand-alone software. More instructions can be found https://github.com/HenriquesLab/NanoJ-Fluidics/wiki/
