# Supplementary material for: Automating multimodal microscopy with NanoJ-Fluidics
Source: Nat Commun. 2019 Mar 15;10:1223. doi: 10.1038/s41467-019-09231-9 (PMC6420627; doi:10.1038/s41467-019-09231-9)
Supplement: Supplementary file 9 — Reporting Summary [file 41467_2019_9231_MOESM9_ESM.pdf]

natureresearch

Corresponding author(s): Romain F. Laine; Christophe Leterrier; Ricardo Henriques

# Reporting Summary

Nature Research wishes to improve the reproducibility of the work that we publish. This form provides structure for consistency and transparency in reporting. For further information on Nature Research policies, see Authors & Referees and the Editorial Policy Checklist.

## Statistical parameters

When statistical analyses are reported, confirm that the following items are present in the relevant location (e.g. figure legend, table legend, main text, or Methods section).

| n/a | Confirmed | |
|---|---|---|
| ☐ | ☒ | The exact sample size (*n*) for each experimental group/condition, given as a discrete number and unit of measurement |
| ☐ | ☒ | An indication of whether measurements were taken from distinct samples or whether the same sample was measured repeatedly |
| ☒ | ☐ | The statistical test(s) used AND whether they are one- or two-sided<br>*Only common tests should be described solely by name; describe more complex techniques in the Methods section.* |
| ☒ | ☐ | A description of all covariates tested |
| ☒ | ☐ | A description of any assumptions or corrections, such as tests of normality and adjustment for multiple comparisons |
| ☒ | ☐ | A full description of the statistics including central tendency (e.g. means) or other basic estimates (e.g. regression coefficient) AND variation (e.g. standard deviation) or associated estimates of uncertainty (e.g. confidence intervals) |
| ☒ | ☐ | For null hypothesis testing, the test statistic (e.g. *F*, *t*, *r*) with confidence intervals, effect sizes, degrees of freedom and *P* value noted<br>*Give P values as exact values whenever suitable.* |
| ☒ | ☐ | For Bayesian analysis, information on the choice of priors and Markov chain Monte Carlo settings |
| ☒ | ☐ | For hierarchical and complex designs, identification of the appropriate level for tests and full reporting of outcomes |
| ☒ | ☐ | Estimates of effect sizes (e.g. Cohen's *d*, Pearson's *r*), indicating how they were calculated |
| ☒ | ☐ | Clearly defined error bars<br>*State explicitly what error bars represent (e.g. SD, SE, CI)* |

*Our web collection on statistics for biologists may be useful.*

## Software and code

Policy information about availability of computer code

| | |
|---|---|
| Data collection | NIS-Elements Ar, NanoJ-Fluidics |
| Data analysis | NIS-A N-STORM analysis module, NanoJ-SRRF, NanoJ-SQUIRREL, ThunderSTORM, Fiji |

For manuscripts utilizing custom algorithms or software that are central to the research but not yet described in published literature, software must be made available to editors/reviewers upon request. We strongly encourage code deposition in a community repository (e.g. GitHub). See the Nature Research guidelines for submitting code & software for further information.

## Data

Policy information about availability of data

All manuscripts must include a data availability statement. This statement should provide the following information, where applicable:

- Accession codes, unique identifiers, or web links for publicly available datasets
- A list of figures that have associated raw data
- A description of any restrictions on data availability

Figure 2, 3, 4, 5, S3, S4, S5, S6, S7 and Movies S1, S2, S3 and S4 have associated raw data that is available upon request. NanoJ-Fluidics follows open-source software and hardware standards, it is part of the NanoJ project (NanoJ-SRRF, NanoJ-SQUIRREL, NanoJ-VirusMapper). The steps to assemble a complete functioning system are described in: https://github.com/HenriquesLab/NanoJ-Fluidics/wiki

# Field-specific reporting

Please select the best fit for your research. If you are not sure, read the appropriate sections before making your selection.

☒ Life sciences ☐ Behavioural & social sciences ☐ Ecological, evolutionary & environmental sciences

For a reference copy of the document with all sections, see nature.com/authors/policies/ReportingSummary-flat.pdf

# Life sciences study design

All studies must disclose on these points even when the disclosure is negative.

| | |
|---|---|
| Sample size | N/A |
| Data exclusions | N/A |
| Replication | All experiments were done at least in duplicate and were always reproducible. |
| Randomization | N/A |
| Blinding | N/A |

# Reporting for specific materials, systems and methods

## Materials & experimental systems

| n/a | Involved in the study | |
|---|---|---|
| ☒ | ☐ | Unique biological materials |
| ☐ | ☒ | Antibodies |
| ☐ | ☒ | Eukaryotic cell lines |
| ☒ | ☐ | Palaeontology |
| ☒ | ☐ | Animals and other organisms |
| ☒ | ☐ | Human research participants |

## Methods

| n/a | Involved in the study | |
|---|---|---|
| ☒ | ☐ | ChIP-seq |
| ☒ | ☐ | Flow cytometry |
| ☒ | ☐ | MRI-based neuroimaging |

## Antibodies

| | |
|---|---|
| Antibodies used | Primary: mouse monoclonal anti-TOM20 (BD Bioscience # 612278), rabbit polyclonal anti-clathrin heavy chain (abcam ab21679), chicken poly-clonal anti-vimentin (BioLegend # 919101) and rat anti-alpha-tubulin (mix of clone YL1/2 abcam # 6160 and clone YOL1/34 Millipore CBL270).<br>Secondary: Goat anti-Mouse (A28174, ThermoFisher), Goat anti-chicken (Abcam, ab7113), Goat anti-Rabbit (A27033, ThermoFisher), Donkey anti-Rat (A18747, ThermoFisher). |
| Validation | All Ab were validated by the manufacturer's and all have been used in multiple publications before:<br>Anti-Tom20 (http://www.bdbiosciences.com/us/reagents/research/antibodies-buffers/cell-biology-reagents/cell-biology-antibodies/purified-mouse-anti-tom20-29tom20/p/612278)<br>Anti-Clathrin (http://www.abcam.com/clathrin-heavy-chain-antibody-ab21679.html)<br>Anti-Vimentin (https://www.biolegend.com/en-us/products/purified-anti-vimentin-antibody-11598)<br>Anti-Tubulin (Ab6160, http://www.abcam.com/tubulin-antibody-yl12-ab6160.html)<br>Anti-Tubulin (YOL1/34, http://www.merckmillipore.com/GB/en/product/Anti-alpha-Tubulin-Antibody-clone-YOL1/34,MM_NF-CBL270-I) |

## Eukaryotic cell lines

Policy information about cell lines

| | |
|---|---|
| Cell line source(s) | COS7 (Figure 4, S5, S6), gift from Professor Mark Marsh (MRC-LMCB, UCL)<br>COS7 (Figure 5 and S7), ATCC<br>hTERT-RPE1 (Figure 2), Clontech<br>HeLa H2B-mCherry/mEGFP-α-Tubulin (HCTG) (Figure 3), kind gift from Daniel Gerlich |

| | |
|---|---|
| Authentication | The cell lines were not authenticated |
| Mycoplasma contamination | Cell lines were negative for mycoplasma |
| Commonly misidentified lines (See ICLAC register) | N/A |
